# Alteration of mRNA and microRNA expression profiles in rat muscular type vasculature in early postnatal development

**DOI:** 10.1038/srep11106

**Published:** 2015-06-15

**Authors:** Dina Gaynullina, Harsh Dweep, Torsten Gloe, Olga S. Tarasova, Carsten Sticht, Norbert Gretz, Rudolf Schubert

**Affiliations:** 1 Cardiovascular Physiology, Centre for Biomedicine and Medical Technology Mannheim, Ruprecht-Karls-University Heidelberg, 68167 Mannheim, Germany; 2 Faculty of Biology, M.V. Lomonosov Moscow State University, 119234, Moscow, Russia; 3 Department of Physiology, Russian National Research Medical University, Ostrovityanova str. 1, 117997 Moscow, Russia; 4 Medical Research Center, University of Heidelberg, D-68167 Mannheim, Germany

## Abstract

The vascular system is characterized by a high degree of plasticity. In particular, functional and structural remodeling of the arterial system takes place during early postnatal development. However, the mechanisms providing such alterations in the rapidly growing organisms are poorly understood, especially for the peripheral vasculature. To explore this, we performed mRNA- and miRNA microarray analysis on muscular type saphenous arteries of young (10–12 days) and adult (2–3 months) rats. Thirty-eight significant pathways (such as oxidative phosphorylation, MAPK signaling, metabolism, cell cycle, DNA replication and focal adhesion) were obtained on differentially regulated genes during postnatal development. Many differentially regulated genes were determined as target- and miRNA-hubs. We also found 92 miRNAs differentially expressed in arteries of young and adult rats. Several significantly regulated pathways were found on these regulated miRNAs. Interestingly, these biological cascades also contain those significantly enriched pathways that were previously identified based on the differently expressed genes. Our data indicate that the expression of many genes involved in the regulation of pathways that are relevant for different functions in arteries may be under the control of miRNAs and these miRNAs regulate the functional, and structural remodeling occurring in the vascular system during early postnatal development.

MicroRNAs (miRNAs) are a class of evolutionarily conserved small non-coding RNAs shown to predominantly negatively regulate gene expression by promoting degradation or suppressing translation of target mRNAs^1^. In a few cases, however, target mRNA activation by miRNAs has also been described^2^. miRNAs modulate various biological functions in animals, plants, and unicellular eukaryotes^3^ by participating in a variety of processes, including cell proliferation, differentiation, growth, apoptosis, stress response, angiogenesis and tumorigenesis^4^.

Initially discovered as regulators of developmental timing in nematodes^5^, miRNAs were found to play a crucial role in the development of mammals from the formation of embryos to the creation of highly specific tissues^6^. Thus, miRNAs were shown to regulate the development of the nervous system^7^, as well as cardiac and skeletal muscles^8^. In the vascular system miRNAs were demonstrated to coordinate its growth in adult animals by affecting neovascularization and angiogenesis^4^. Additionally, their role in the modulation of vascular smooth muscle cell phenotype was revealed^9^.

Importantly, in the mature vascular system, smooth muscle cell-specific deletion of Dicer, an important enzyme regulating miRNA processing, causes a dramatic reduction of blood pressure and a loss of vascular contractile function^10^ pointing to a prominent role of miRNAs in the maintenance of vascular contractility.

Of note, vascular contractility undergoes changes during early postnatal development of the circulatory system reflecting its high degree of plasticity during maturation. This enables an appropriate blood supply of fast growing organs and tissues, and is accompanied by dramatic changes of hemodynamic parameters, including an increase of peripheral vascular resistance and blood pressure^11^. Nowadays, studies about the mechanisms and regulation of vascular functioning during early postnatal ontogenesis have attracted growing attention, because of an increased occurrence of obesity, insulin resistance and type II diabetes in childhood^12^. Moreover, widespread chronic diseases in adulthood, e.g. endothelial dysfunction and hypertension, may have their origin in inappropriate cardiovascular development in the postnatal period^13^. Interestingly, first studies appeared showing the involvement of miRNAs in developmental processes in the circulatory system, like senescence and aortic aneurism^14^. Recently, a study reported changes in miRNA expression also during postnatal development in rat aorta^15^.

In the circulatory system a large degree of functional diversity has been observed. The aorta is a conduit vessel responsible for the transformation of a discontinuous into a more continuous flow but is not involved in blood flow distribution and blood pressure regulation. In this vessel, changes in smooth muscle contractility affect mostly vessel wall stiffness and not so much vessel diameter. In contrast, peripheral vessels, especially highly innervated muscular type arteries, contribute considerably to blood flow distribution and blood pressure regulation. Importantly, the functional differences between these vessel types are reflected by remarkable differences in contractile mechanisms, including the differences in alpha_1_-adrenoceptor populations, as well as in Ca^2+^-signaling and Ca^2+^-sensitizing mechanisms^16^. For example, in rat small muscular type arteries the α_1_-adrenergic contraction invokes protein kinase C activation, but not Rho-kinase, while in rat aorta it is mediated by Rho-kinase and is not affected by protein kinase C^16^. These differences in contractile mechanisms may be the result of different developmental programs governed by, for example, miRNAs. However, whether indeed developmental changes in miRNA expression are different in different vessels is unknown. Thus, this study tested the hypothesis that mRNA and miRNA expression profiles change in the muscular type rat saphenous artery during early postnatal development and that these changes are different compared to conduit arteries.

To address this question, first, we performed a high-throughput study (using m- and miRNA microarrays) to profile changes in mRNA and miRNA expression in muscular type arteries between young (10–12 day old) and adult (2–3 months old) rats. Second, we accomplished a bioinformatics analysis including microarray data analysis, pathways and gene ontology (GO) terms enrichment to determine significant genes, miRNAs and biological cascades. In addition, we employed a meta-analysis for miRNA-target predictions to identify possible interactions between significantly regulated genes and miRNAs. Furthermore, we carried out miRNA binding site enrichment analysis to obtain significantly overrepresented candidates and predicted miRNAs that could regulate significant pathways. Third, we compared these postnatal changes in miRNA expression profile with miRNA changes (from day one to 12 month old) in a conduit artery type obtained from the literature. Figure 1 shows the workflow of the current investigation. The findings of the present study support our hypothesis and suggest that miRNAs may be involved in the genetic regulation of muscular type vasculature in early postnatal development.

## Results

### Bioinformatics analysis of mRNA microarray data

To elucidate in a high-throughput manner which functional patterns (pathways) are differentially expressed in muscular type vasculature during early postnatal development, microarray profiling was performed and 8,266 differentially expressed genes were identified (p-value < 0.01 after FDR correction) between adult and young animals. Of these, expression of 4,425 genes was higher in arteries of adult rats (i.e. up-regulated during postnatal development) and the expression of the remaining 3,841 genes was lower in arteries of adult animals (i.e. down-regulated during postnatal development) (Table S1). The top 40 (20 most up-regulated and 20 most down-regulated) regulated genes in arteries of adult versus young animals are depicted in Fig. 2. These genes are known to be associated with diverse signaling pathways such as cell adhesion, proteolysis, calcium signaling, amino acid transport and metabolism. Moreover, several transcription factors (such as Cebpb, Junb, Mitf, Stat1, Id1, Zeb1, Pparα), chromatin remodeling factors (e.g., Hdac2, Hdac5, Vps24 and Bmi1) and cofactors (including Ankrd1, Ywhab, Nab1 and Nfkbib) were found to be differentially expressed in vessels of animals of different age (Table S1). These genes have previously been shown to play a pivotal role in cardiovascular development and physiology^17^ by modulating diverse biological processes such as cell cycle, proliferation, apoptosis, DNA replication, focal adhesion, ECM-receptor interactions and metabolic pathways.

Further, 23 and 15 biological pathways were found statistically significant (p-value < 0.05 after BH correction) for up- and down-regulated genes, respectively. The most relevant up- and down-regulated pathways (such as oxidative phosphorylation, MAPK signaling, metabolism, cell cycle, DNA replication, ECM-receptor interaction, focal adhesion and steroid biosynthesis) are shown in Table 1. Furthermore, several gene ontologies (GO) (biological processes (BP), molecular functions (MF) and cellular components (CC)) were also obtained on significantly regulated genes (Table S2).

Among different pathways regulated during arterial postnatal development, the vascular smooth muscle contractile pathway is of special interest because it reflects one of the most essential arterial functions, especially of muscular type arteries, the regulation of vascular resistance and blood pressure. Figure S1, based on the KEGG database, shows that (i) the expression level of most members of this pathway changes during early postnatal development and (ii) the vast majority of the altered genes are up-regulated with age.

In addition, Ca^2+^ ions are one of the most important intracellular regulators and messengers in many tissues, in particular in vascular smooth muscle. Interestingly, many members of the Ca^2+^-signaling pathway change their expression in muscular type arteries during early postnatal development (Figure S2 and Table S3), including voltage-dependent Ca^2+^-channels, ryanodine and inositol 1,4,5-trisphosphate receptors, pointing to significant alterations not only in the smooth muscle contractile machinery in general, but in particular in Ca^2+^ homeostasis during postnatal development.

Next, we utilized the comparative platform of the miRWalk database (http://mirwalk.uni-hd.de) to find out the possible regulation of miRNAs within differently expressed genes in muscular type arteries during postnatal development. Interestingly, numerous possible miRNA binding sites were detected within up- (359,681 interactions; ~98 miRNA binding sites/gene) and down-regulated (323,669 interactions; ~104 miRNA binding sites/gene) genes. For example, Pparα (regulates metabolic pathways) was found to harbor putative binding sites for miR-19b/351 (5 algorithms), miR-17-5p (3 algorithms), miR-214 and -503 (2 algorithms). Also, many up-regulated transcription factors, chromatin modulators and cofactors (such as Tbl1x, Bcl6, Runx1, Klf6, Foxs1, Chmp3, Hdac5, Smarca2, Nab1, Fhl5) were identified to have possible binding sites for multiple miRNAs including miR-18a, -19b, -20a, -127 and -214. Similarly, other miRNAs (e.g., miR-1, -29a/b/c, -210 and -133a/b) were also predicted to target several down-regulated genes encompassing Ets1, Hoxa2, Igf2, Tp53, Foxa2, Gata2, Cbx1 and Mtf2. Moreover, many significantly regulated genes and predicted miRNAs were determined as target-hubs (n = 5,624; targets with at least 15 binding sites) and miRNA-hubs (n = 658; miRNAs targeting at least 15 genes). Thereafter, all of these predicted miRNAs were subjected to an overrepresentation analysis to determine only those miRNAs that are significantly enriched for their binding sites within our regulated genes compared to all known genes of the rat. In total, 311 and 466 miRNAs were significantly enriched for their binding sites within up- and down-regulated genes, respectively (Table S4). Our findings are in accordance with previous investigations suggesting that each of miRNAs having the potential to anneal with hundreds of targets^18^.

### Validation of mRNA expression changes using qPCR

In order to validate the results obtained with mRNA microarrays we determined the expression of several differently expressed genes using qPCR. In particular, we tested the expression of several highly regulated genes, as detected by the microarray data shown in Fig. 2 and several members of the voltage-gated potassium channel family, that are important for the regulation of vascular function (Fig. 3A). These data were compared with the microarray data (Fig. 3B). The qPCR analysis confirmed that the expression of Plekhb2, Fgf1, Dpep1, Kcna2 and Kcna5 is higher in arteries of adult compared to young animals; the expression of Postn and Gnas is higher in arteries of young rats. No change in expression was observed for Kcna7. In addition, our previously published findings demonstrate a higher expression of eNOS in arteries of young animals using qPCR^19^ and this is similar to the data obtained on mRNA microarray. Altogether, our data on mRNA microarray and qPCR are in a good agreement, indicating high level of data reproducibility.

### Bioinformatics analysis of miRNA microarray data

Our meta-analysis of binding site predictions on significantly regulated genes suggests that many miRNAs could play a possible role in the genetic regulation of muscular type arteries during postnatal development. Hence, miRNA microarray profiling was performed to examine the differential expression of miRNAs between adult and young animals in which a total of 92 (30 up- and 62 down-regulated) miRNAs were significantly different (Fig. 4). We further wanted to determine the possible role of these 92 miRNAs in different signaling cascades; therefore, miRWalk was utilized for gathering the information on putative target genes and these target genes were then interrogated against all known KEGG pathways. A total of 118 (such as steroid biosynthesis and cell cycle) and 144 (e.g., TCA cycle and MAPK signaling) significantly regulated pathways were found on 30 up- and 62 down-regulated miRNAs, respectively (Table S5). Interestingly, these biological cascades also contain those significantly enriched pathways that we previously identified based on the differently expressed genes. These results suggest a preliminary confirmation of our hypothesis according to which different miRNAs could be involved in the regulation of pathways that are relevant in muscular type arteries during early postnatal development.

### Common findings between m- and mi-RNA microarray data

Accumulating studies conducted over the last decades suggest that the mechanism of action of most miRNAs is to negatively regulate the expression of their targets via annealing in a sequence specific manner 1. Therefore, miRNA-gene interactions should be manifested in inverse correlations of their expression levels. For example, if expression levels of one or more miRNAs are increased then expressions of their targets should be decreased and vice-versa. To find out such interactions in our study, the results of m- and mi-RNA microarray profiling were compared in which 3,269 and 2,505 candidates were commonly identified for up-genes/down-miRNAs and down-genes/up-miRNAs, respectively (Table 2; Table S6). Interestingly, 14 (up-genes/down-miRNAs) and 13 (down-genes/up-miRNAs) significantly regulated pathways were found overlapping between m- and miRNA microarray data (Table S6).

Furthermore, 54 (7 up- and 47 down-regulated) out of 92 miRNAs (obtained from miRNA microarrays) were significantly enriched for their binding sites within regulated genes (Tables S6 and S7).

### Validation of potential miRNAs using qPCR

m- and mi-RNA microarrays have already been proven to be potential tools for profiling the global expression of genes and miRNAs; however, nowadays it has become common practice to utilize a second quantitative measure for validating the microarray data. The qPCR technique has been extensively used for such validations. Thus, we performed qPCR experiments for 8 potential candidate miRNAs: miR-134, -1, -194, -195, -347, -29a, -29b and -29c. These miRNAs were shown to be strongly regulated during early postnatal development as described above. The results of the qPCR experiments (Fig. 5) show a large increase of miR-29a, -29b, -29c, -1, -194, -195, -347 and a decrease of miR-134 expression during early postnatal development in muscular type vasculature corroborating the microarray data.

### Possible interactions of the 8 validated miRNAs with regulated genes/pathways

To determine the possible interactions of these 8 miRNAs with regulated genes and their regulatory pathways, target genes and significantly enriched pathways were extracted from miRWalk. We ended up with 4,311, 4,687, 4,221, 4,961, 4,263, 3,662, 3,692, and 3665 putative target genes for miR-134, -1, -194, -195, -347, -29a, - 29b and -29c, respectively (Fig. 4). On comparing, 2,100, 2,277, 2,101, 2,457, 2,089, 1,819, 1,838, and 1,820 candidate genes were found common between the target-genes identified by miRWalk and the regulated ones resulting from mRNA microarray profiling (Fig. 4). A total of 67 significantly enriched pathways, including several biologically important ones such as metabolism, Wnt signaling, endocytosis, circadian rhythm, cell cycle, MAPK signaling and focal adhesion, were found only for the 8 validated miRNAs (Figure S3 and Table S7). Furthermore, 33 out of 67 enriched pathways were identified as commonly predicted pathways based on at least 2 different miRNAs (Table S8). Table S8 displays an overview of significantly enriched pathways predicted by at least 2 different miRNAs.

Interestingly, 24 biological cascades (e.g., up-regulated: metabolic pathways, MAPK signaling, VEGF signaling, and calcium signaling; down-regulated: focal adhesion and cell cycle) out of these 67 are among those pathways that were also obtained significantly over-represented during the analysis of regulated genes using mRNA microarray data.

## Discussion

We herein performed a large-scale investigation encompassing mRNA and miRNA microarrays profiling, meta-analysis for miRNA-target interaction predictions, pathways, gene ontologies and miRNA binding site over-representation analysis to shed light on the alteration of genes and miRNAs expression in muscular type vasculature in early postnatal development, to explore the signaling cascades engaged with these genes and miRNAs and to find out the potential role of miRNA-mediated regulation of these genes and their pathways.

Microarray profiling showed that 8,266 genes were differentially expressed during postnatal development in muscular type vasculature (4,425 up-regulated, 3,842 down-regulated) that are associated with diverse signaling pathways, gene ontologies, transcription factors, chromatin remodeling factors and cofactors. These genes have previously been shown to play a pivotal role in cardiovascular development and physiology^17^ by modulating diverse biological processes such as cell cycle, proliferation, apoptosis, DNA replication, focal adhesion, ECM-receptor interactions and metabolic pathways. This huge pool of regulated genes supports the idea of complex functional changes occurring in the arterial system during early postnatal development.

Instructive examples of these functional changes are the smooth muscle contractile pathway and the Ca^2+^-signaling pathway. These pathways underlie the contractile function of blood vessels that is responsible for the regulation of blood flow distribution and blood pressure. Indeed, an increase in peripheral resistance and blood pressure level within the first weeks after birth has been observed^11^. Our data show postnatal alterations in the expression levels of many participants of the vascular smooth muscle cell contractile pathway (Figure S1). These extensive postnatal alterations in gene expression are supported by our previous functional findings on these arteries showing that the contractile responses of muscular type arteries from young and adult animals are differently regulated. While in arteries of adult animals the rapid contractile response is heavily Ca^2+^-dependent, in arteries of young animals the contractile machinery is sensitized to Ca^2+^ and can develop the contraction in response to only a slight elevation of the intracellular Ca^2+^ concentration^20^. These functional alterations during early postnatal development were accompanied by changes in protein expression^20^ that often coincided with the changes in mRNA expression observed in the present study (for example, for ERK1/2, MYPT1 or caldesmon), but sometimes did not correspond to the changes in mRNA expression observed in the present study (opposite changes or no changes were observed, e.g. for Rho-kinase, p38 MAPK). In addition, the data of the present study show that the arteries of adult animals exhibit a higher expression level of many voltage-dependent Ca^2+^ channels, pointing to the probability of larger Ca^2+^ entry into the smooth muscle of adult in comparison to young animals (Table S3). Moreover, most of the ryanodine and inositol 1,4,5-trisphosphate receptors are up-regulated during postnatal development, altogether suggesting an increased role of Ca^2+^-dependent mechanisms in arteries of adult animals. Again, these findings are supported by our previous functional data showing that arteries of young animals contract in response to vasoconstrictors almost without detectable changes of the intracellular calcium concentration whereas contractions of arteries from adult animals are associated with a prominent increase of intracellular calcium^20^. In conclusion, the present as well as previous findings, encompassing data from the molecular level (mRNA and protein expression) to the level of an intact organ (contractility and intracellular calcium of isolated arteries) demonstrate an impressive amount of functional remodeling in the circulatory system during postnatal development.

Despite an increasing amount of evidence for functional remodeling in the circulatory system, the mechanisms regulating this process are largely unknown. There is growing evidence for an important functional role of a cluster of regulatory molecules, miRNAs, in diverse remodeling processes. Indeed, based on the gene expression data discussed in the previous paragraph and using a bioinformatics approach, 311 and 466 miRNAs were significantly enriched for their binding sites within up- and down-regulated genes, respectively. These findings are in accordance with previous investigations suggesting that each miRNA has the potential to anneal with hundreds of targets^18^. Moreover, microarray profiling showed that 92 miRNAs (30 up- and 62 down-regulated) were differently expressed during postnatal development in muscular type vasculature. The difference in the expression level of some miRNAs in muscular type arteries of young and adult animals was confirmed experimentally by qPCR. One of the most strongly altered miRNA during postnatal development is the miRNA-29 family (miRNA-29a, -29b and -29c), that is associated with different signaling cascades. Interestingly, these cascades also contain those significantly enriched pathways that were previously identified based on differently expressed genes.

In contrast to muscular type vasculature, conduit arteries were shown to possess a smaller number of altered miRNAs (62 versus 92 in the muscular type artery)^15^. Among them, only 28 miRNAs changed in the same direction during postnatal development in both types of arteries, one miRNA changed in the opposite direction (miR-1). Thus, one may conclude, that the remaining 33 miRNAs are specific for the postnatal development of conduit type of arteries, while 63 are specific for the development of muscular type vasculature. These data indicate that two functionally different types of arteries have a number of common miRNAs responsible for postnatal development, but also possess their own specific miRNAs, that are probably crucial for the development of specific functional aspects in the respective type of vasculature.

Notably, previously published studies provide some information about possible interactions between developmentally regulated miRNAs and genes, for example, regarding the miR-29 family. Thus, miR-29 has been shown experimentally to interact with structural genes, such as collagen and elastin in conduit arteries^14^,^15^,^21^. Similarly, in the present study on muscular type arteries 23 genes encoding collagen were found to be differentially expressed on the mRNA level, 19 of them showed a higher expression in young animals and only 4 - in arteries of adult rats. In addition, the expression of elastin was stronger in arteries of young rats, a finding in accordance with previous data obtained from aortas of 1-day-old and 12-month-old rats^15^. Remarkably, the main direction of changes of these genes (decrease in mRNA expression during maturation) is in agreement with the developmental increase of miR-29 expression under the assumption that miRNAs negatively regulate gene expression. Therefore, the finding of an established interaction between miR-29 and structural genes reported in the previous literature and our observation of differently expressed miR-29 and mRNA for collagen and elastin strongly suggest a possible interaction between these miRNAs and the regulated structural genes in the vessel investigated in our study.

## Conclusions

In conclusion, our data together with published findings indicate that the regulation of expression of many genes involved in the regulation of pathways that are relevant in arteries during early postnatal development may be under the control of miRNAs. In addition, our findings support the idea that one miRNA may affect the expression of many mRNAs.

## Methods

### Animals

In this study, 10–12 days old (“young”) and 2–3 months old (“adult”) male Wistar rats obtained from Janvier were used. All animal experiment protocols were *approved* by the US National Institutes of Health (NIH Publication No. 85–23, revised 1996), German Animal Law and regulations at Heidelberg University. All the methods were carried out in *accordance* with the approved guidelines. Rats were anesthetized with CO_2_ inhalation and then sacrificed by decapitation.

### RNA isolation for microarray analysis

One sample consisted of either four saphenous arteries obtained from 2 young animals or two saphenous arteries obtained from one adult animal. There were four samples in each group (young and adult animals). After isolation arteries were quickly frozen in liquid nitrogen and stored at -80 °C. Total RNA for mRNA microarrays was extracted using Master Pure RNA Purification Kit (EPICENTRE). Total RNA for miRNA microarrays was extracted using miRNeasy Mini Kit (Qiagen).

### Affymetrix mRNA microarray

RNA quality was tested by capillary electrophoresis on an Agilent 2100 bioanalyzer (Agilent Technologies, USA). Only samples with a RNA integrity number larger than 7 were used for further analysis. cDNA, and cRNA synthesis, labeling and hybridization were carried out according to the manufacturer (Affymetrix, USA). Gene expression profiling was performed using Affymetrix Rat Gene microarrays (n = 8; GeneChip® Rat Gene 1.0). mRNA microarray data were deposited in Gene Expression Omnibus database (accession no. GSE60961).

### Quantitative mRNA-PCR

Total RNA was isolated from arteries of young and adult animals, as described above (see *RNA isolation for mRNA microarray analysis*). Quantitative PCR was performed in 96-well plates (Ligth Cycler 480 PCR system, Roche) using MAXIMA SYBR Green qPCR Master Mix (Fermentas). GAPDH was used for normalization. Primer sequences (5’–3’) for Plekhb2 (forward: CCCCTGAGGTCTACGGCTAT, reverse: GGTCACTGTCACTGTCTCGG), Fgf1 (forward: GTTTGCAGAGGACGCTGGAT, reverse: GCGTTCAAGACAGGTCAGATG), Dpep1 (forward: CTCTCTCTGGGTTTCAGCCG, reverse: AAGTCGTTGTGCCCGTCAAT), Postn (forward: TGCAAAAAGACACACCTGCAA, reverse: CCGAAGTCAATGGGGCTCTT), Gnas (forward: TTGGCCCAGCCGAAGAAAT, reverse: GCTCCAAAATCGCTGGGTTG), Kcna2 (forward: TCAAGTCGTGCGCTGTGCCC, reverse: CCTTTGGCTGGCAGGCAGGA), Kcna5 (forward: GGCTATCTGCTGAGGATGAG, reverse: CGGTCGAAGAAGTATTCATTTCTC), Kcna7 (forward: CTGGTCTCCATCGTGGTCTT, reverse: GGGACTGGAGCCATTGAG), GAPDH (forward: CACCAGCATCACCCCATT, reverse: CCATCAAGGACCCCTTCATT). Efficiencies of the primers were determined directly from the fluorescence data of each PCR. Statistical significance was determined using the unpaired t-test. Statistical significance was reached at P < 0.05.

### Affymetrix miRNA microarray

Quality of RNA was tested as described above. 1 μg of total RNA was Biotinylated using FlashTag Biotin RNA Labeling Kit according to the manufacturer’s protocol (Genisphere LLC). Labeled product was hybridized to Affymetrix miRNA microarrays (n = 8; GeneChip® miRNA 3.0) followed by washing, staining and scanning as described elsewhere^22^. miRNA microarray data were deposited in Gene Expression Omnibus database (accession no. GSE60962).

### Quantitative miRNA-PCR

Total RNA was isolated from arteries of young and adult animals, as described above (see *RNA isolation for miRNA microarray analysis*). Reverse transcription and quantitative PCR were performed, respectively, using Universal cDNA Synthesis Kit II and ExiLENT SYBR® Green master mix together with MicroRNA LNA PCR primer sets from Exiqon, according to the protocols of the manufacturer. The amplification was done in 96 well plates (Ligth Cycler 480 PCR system, Roche). The small nuclear RNA U6 gene was used for normalization. Efficiencies of the primers were determined from the fluorescence data of every PCR. Statistical significance was determined using the unpaired t-test. Statistical significance was reached at P < 0.05.

### Bioinformatics analysis of m- and mi-RNA microarrays data

After preliminary evaluation (such as minimization of bad quality intensity signals, quality control and scatter plot analysis), the quantile normalization method was applied to normalize the raw fluorescence intensity values (GSE60963) and differential gene expression was calculated by one-way analysis of variance (ANOVA)^23^ using the software package SAS JMP7 Genomics 4 (SAS Institute, Cary, NC, USA). A false-positive rate of α = 0.05 with false discovery rate (FDR) correction was taken as the level of significance. m- and mi-RNA microarrays were annotated using custom chip definition file (CDF, version 14 with Entrez GeneIDs based gene definitions)^24^ and standard miRNA annotation file (provided by Affymetrix), respectively. The data were filtered for genes by considering expression changes (between adults and young animals i.e. AD-NB) either greater than 1-fold or less than 1-fold with an adjusted p-value < 0.01 and for miRNAs whose expression changed 1.5-fold (with p < 0.001) in adult compared with young (control) animals.

**Figure 1:**
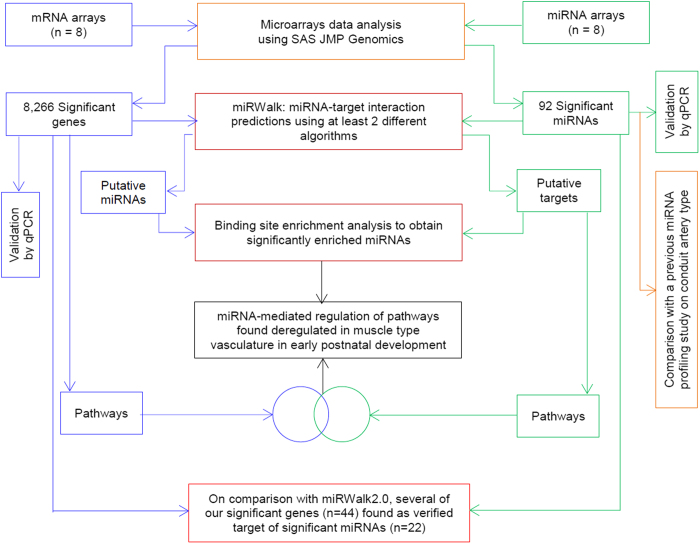
Schematic workflow to study differentially regulated mRNAs, miRNAs and pathways in muscular type vasculature.

**Figure 2:**
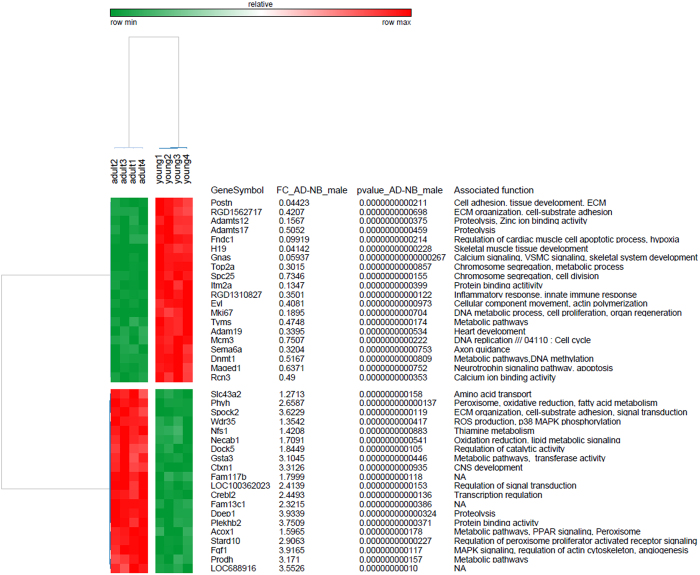
Differential expression of top 40 genes in arteries of adult and young animals. The heatmap was produced by clustering the data matrix of top 40 genes using Pearson correlation. The gene clustering tree is shown on the left and the sample clustering tree is shown on the top. The other information such as gene symbol and associated function are given on the right. The samples are broadly divided into two groups, adult and young. The color scale shown at the top illustrates the relative expression level of the indicated genes across all samples.

**Figure 3:**
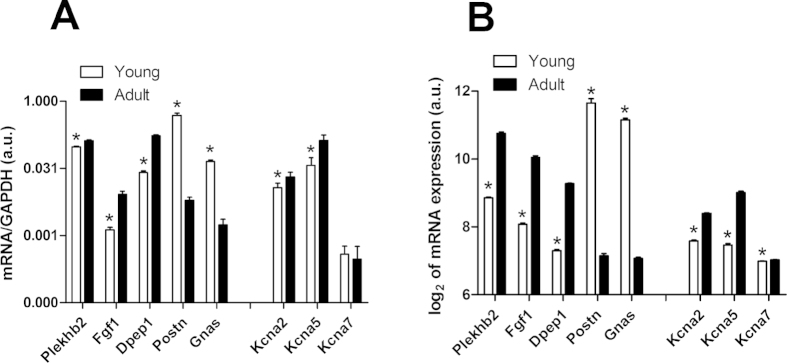
Differential expression of 8 mRNAs in arteries of adult and young animals. The expression level of 8 mRNAs in arteries of young and adult animals was determined by qPCR (panel A, n = 6; 7 or 8 for each mRNA in every group). For comparison, data obtained using the mRNA microarray technique for the same mRNAs are presented in panel B. * p < 0.05.

**Figure 4:**
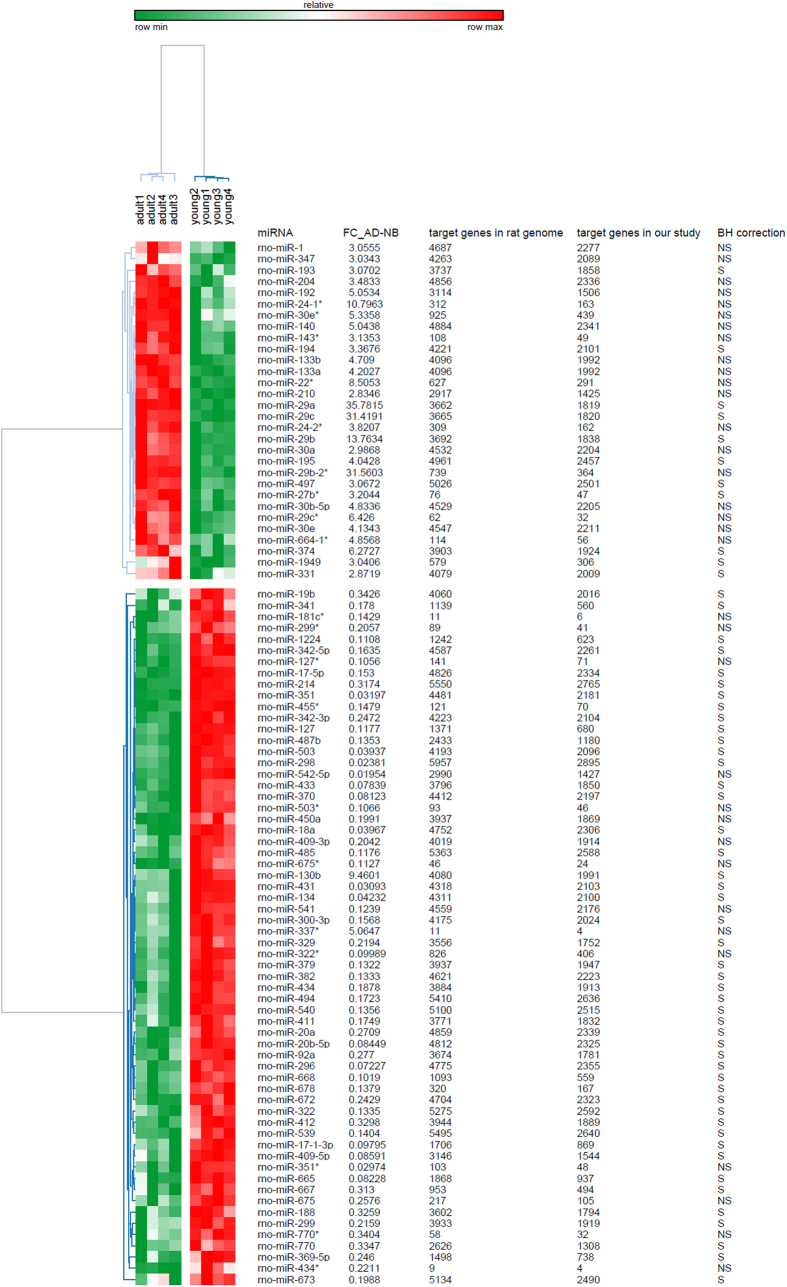
Differential expression of 92 miRNAs in arteries of adult and young animals. The heatmap was produced by clustering the data matrix of 92 miRNAs using Pearson correlation. The miRNA clustering tree is shown on the left and the sample clustering tree is shown on the top. The other information such as miRNA name, number of possible targets in rat genome and in our animals, and BH correction (‘S’ and ‘NS’ denote significant, and non-significant, respectively) are given on the right. The samples are broadly divided into two groups, adult and young. The color scale shown at the top illustrates the relative expression level of the indicated genes across all samples.

**Figure 5:**
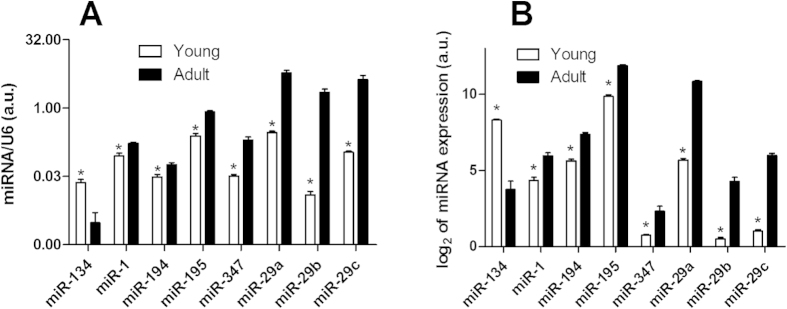
Differential expression of 8 miRNAs in arteries of adult and young animals. The expression level of 8 miRNAs in arteries of young and adult animals was determined by qPCR (panel A, n = 6 for each miRNA in every group). For comparison, data obtained using the miRNA microarray technique for the same miRNAs are presented in panel B. * p < 0.05.

Significant candidates were classified into two groups (up- and down-regulated genes, i.e. with higher and smaller expression level in adult compared to young animals, respectively) and these groups were then used to determine significantly enriched KEGG pathways^25^. In order to carry out a pathway enrichment analysis, first, all known rat pathways, their identifiers and gene-sets were gathered from KEGG database. Second, this information was preprocessed and complied into a two columns file. Third, this complied file was then loaded into R-statistical package (version 3.0.2) to generate “R-dataset” by using “read.table” function. In a next step, the significantly up- and down-regulated genes were interrogated against the R-dataset with the help of a customized “R script” using Fisher’s exact test with the Benjamini and Hochberg (BH) method with 5% level of significance^22^. Afterwards, the comparative platform of miRWalk^26^ was employed for conducting a meta-analysis to identify the possible miRNA binding sites for these two groups. miRWalk documents putative miRNA-target interactions resulting from 10 different prediction datasets: DIANA-microT, miRanda, miRWalk, miRDB, RNA22, RNAhybrid, PicTar4, PicTar5, PITA and Targetscan. Information on these datasets and their searching algorithms has previously been provided in 27. Integrating the possible miRNA binding sites from different prediction programs have previously been considered for reducing the false positive targets^22^,^27^,^28^,^29^,^30^. Therefore, the gene-miRNA interactions predicted with at least 2 different algorithms were separated and subjected to an overrepresentation analysis for identifying miRNAs that are significantly enriched for their binding sites within up- and down-regulated genes (Fig. 1) compared to all known genes of rat.

Similarly, target genes prediction meta-analysis, pathway and binding site enrichment analysis were accomplished for significantly up- and down-regulated miRNAs (Fig. 1).

## Additional Information

**How to cite this article**: Gaynullina, D. *et al.* Alteration of mRNA and microRNA expression profiles in rat muscular type vasculature in early postnatal development. *Sci. Rep.* **5**, 11106; doi: 10.1038/srep11106 (2015).

## Supplementary Material

Supplementary Information

Supplementary Information

Supplementary Information

Supplementary Information

Supplementary Information

Supplementary Information

Supplementary Information

Supplementary Information

## Figures and Tables

**Table 1:** **Differentially regulated pathways on significantly up- and down-regulated genes**. The last three columns display gene sets probability values without p-value adjustment, fold enrichment score and p-value adjustment (after BH correction).

**Pathway**	**p-value**	**Fold enrichment**	**BH correction**
***Pathways associated with significantly up-regulated genes***			
Oxidative phosphorylation	1.10E-11	3.13	1.16E-09
Cardiac muscle contraction	0.0001	2.34	0.003
Metabolic pathways	0.0003	1.27	0.006
Peroxisome	0.0005	2.25	0.01
Phagosome	0.0007	1.68	0.01
VLI degradation	0.0008	2.63	0.01
Pentose phosphate	0.0009	3.95	0.01
TCA cycle	0.001	3.34	0.01
Glycolysis/Gluconeogenesis	0.002	2.06	0.02
Phosphatidylinositol	0.002	2.03	0.03
Pyruvate metabolism	0.003	2.58	0.03
Propanoate metabolism	0.003	2.78	0.03
MAPK signaling	0.004	1.44	0.04
Insulin signaling	0.004	1.67	0.04
Proteasome	0.005	2.27	0.05
***Pathways associated with significantly down-regulated genes***			
Cell cycle	2.63E-13	3.94	2.76E-11
Axon guidance	1.58E-10	3.31	1.11E-08
DNA replication	4.19E-06	5.07	0.0001
Notch signaling	5.33E-06	4	0.0001
ECM-receptor interaction	3.38E-06	3.12	0.0001
Adherens junction	1.63E-05	2.93	0.0004
Focal adhesion	2.45E-05	1.97	0.0005
Steroid biosynthesis	0.001	5.15	0.02

**Table 2:** **Summary of bioinformatics analyses on m- and mi-RNA microarrays**. This table shows the results obtained from m- and mi-RNA microarrays data and their overlapped findings. ‘1’ and ‘2’ denote the common genes among up-genes/down-miRNAs and down-genes/up-miRNAs, respectively. Similarly, ‘3’ and ‘4’ indicate the overlapping enriched pathways obtained from up-genes/down-miRNAs and down-genes/up-miRNAs, respectively, whereas ‘5’ and ‘6’ depicts the commonly significantly enriched miRNAs resulting from up-genes/down-miRNAs and down-genes/up-miRNAs, respectively.

**Pathway**	**Up-regulated**	**Down-regulated**
***mRNA microarrays profiling results***		
Genes	4,425	3,841
Pathways	23	15
Enriched putative miRNAs	311	466
***miRNA microarrays profiling results***		
miRNAs	30	62
Pathways	118	144
Enriched miRNAs	11	47
***Common results between m- and mi-RNA microarrays profiling***		
Genes	3,269^1^	2,505^2^
pathways	14^3^	13^4^
Enriched miRNAs	38^5^	11^6^
