# Supplementary material for: Alteration of mRNA and microRNA expression profiles in rat muscular type vasculature in early postnatal development
Source: Sci Rep. 2015 Jun 15;5:11106. doi: 10.1038/srep11106 (PMC4466593; doi:10.1038/srep11106)
Supplement: Supplementary Information [file srep11106-s1.doc]

**Supplement to**

**Alteration of mRNA and microRNA expression profiles in rat muscular type vasculature in early postnatal development**

Dina Gaynullina^§,1,2,3^, Harsh Dweep^§,4^, Torsten Gloe^1^, Olga S. Tarasova^2^, Carsten Sticht^4^, Norbert Gretz^4^, Rudolf Schubert^1*^

^1^Cardiovascular Physiology, Centre for Biomedicine and Medical Technology Mannheim, Ruprecht-Karls-University Heidelberg, 68167 Mannheim, Germany;

^2^Faculty of Biology, M.V. Lomonosov Moscow State University, 119234, Moscow, Russia;

^3^Department of Physiology, Russian National Research Medical University, Ostrovityanova str. 1, 117997 Moscow, Russia;

^4^Medical Research Center, University of Heidelberg, D-68167 Mannheim, Germany;

^§^Authors contributed equally to this work.

^*^Corresponding author

**Supplementary Figures 1-3**

**Supplementary Tables 1-8**

**Figure S1. Differential expression of vascular smooth muscle contraction pathways based on KEGG database.** Red – expression is higher in arteries of adult animals (i.e. up-regulated), green – expression is higher in arteries of young animals (i.e. down-regulated), white – expression is not different between two age groups.

**Figure S2. Differential expression of members of the Ca^2+^ signaling pathway based on KEGG database.** Red – expression is higher in arteries of adult animals (i.e. up-regulated), green – expression is higher in arteries of young animals (i.e. down-regulated), white - expression is not different between two age groups.

**Figure S3. Differential expression of only those regulated genes that are predicted targets of**

**all 8 verified miRNAs**. The heatmap was produced by clustering the data matrix of regulated

members using Pearson correlation. The gene clustering tree is shown on the left and the sample

clustering tree is shown on the top. The other information such as EntrezID, gene symbol, fold

change and significantly pathways are given on the right. The samples are broadly divided into

two groups, adult and young. The color scale shown at the top illustrates the relative expression

level of the indicated genes across all samples.

**Table S1. Significantly regulated genes found in the adult animals**. This table shows significantly regulated genes on the Affymetrix mRNA microarrays obtained from SAS analysis. A threshold cut-off value was set to 1fold change which indicates that the expression of a given gene is uniform in both adult and young rats. The fold change values greater than 1 indicate up-regulated genes, whereas, the remaining fold change values describe down-regulated genes in adult animals.

**Table S2. Significantly regulated gene ontologies obtained on regulated genes**. This table shows significantly enriched gene ontology terms: biological processes (GOBP), molecular function (GOMF) and cellular components (GOCC) found on up- and down-regulated genes of adult animals.

**Table S3. Developmental changes in the expression levels of voltage-dependent Ca^2+^ channels, ryanodine and inositol 1,4,5-trisphosphate receptors.**

**Table S4. Significantly overrepresented miRNAs within regulated genes**. This table shows the miRNAs obtained significantly enriched for their binding sites within up- and down-regulated genes of adult animals. “Star (*)” symbol denotes opposite arm of a mature miRNA.

**Table S5. Significantly enriched pathways on 92 regulated miRNAs.** This table shows significantly enriched pathways obtained from the target-genes of 92 regulated miRNAs.

**Table S6. Information on mRNA and miRNA microarrays quality control analysis.**

**Table S7. Significantly enriched pathways on 8 validated miRNAs.** This table shows significantly enriched pathways obtained from the target-genes of 8 verified miRNAs.

**Table S8. Significantly enriched pathways identified by at least 2 validated miRNAs.** This table shows significantly enriched pathways predicted by at least 2 validated miRNAs.

**Figure S1.**


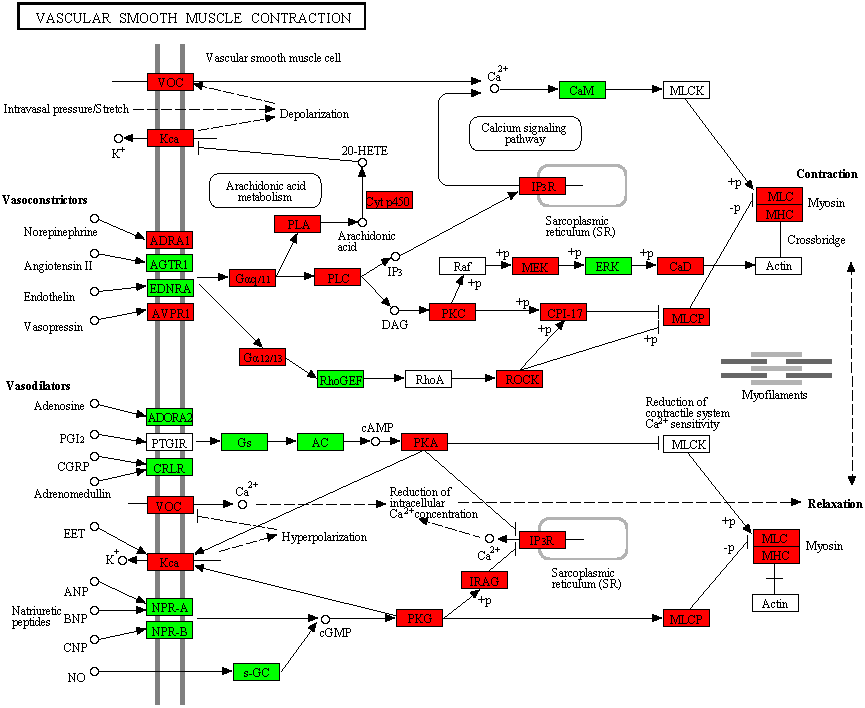


**Figure S2.**


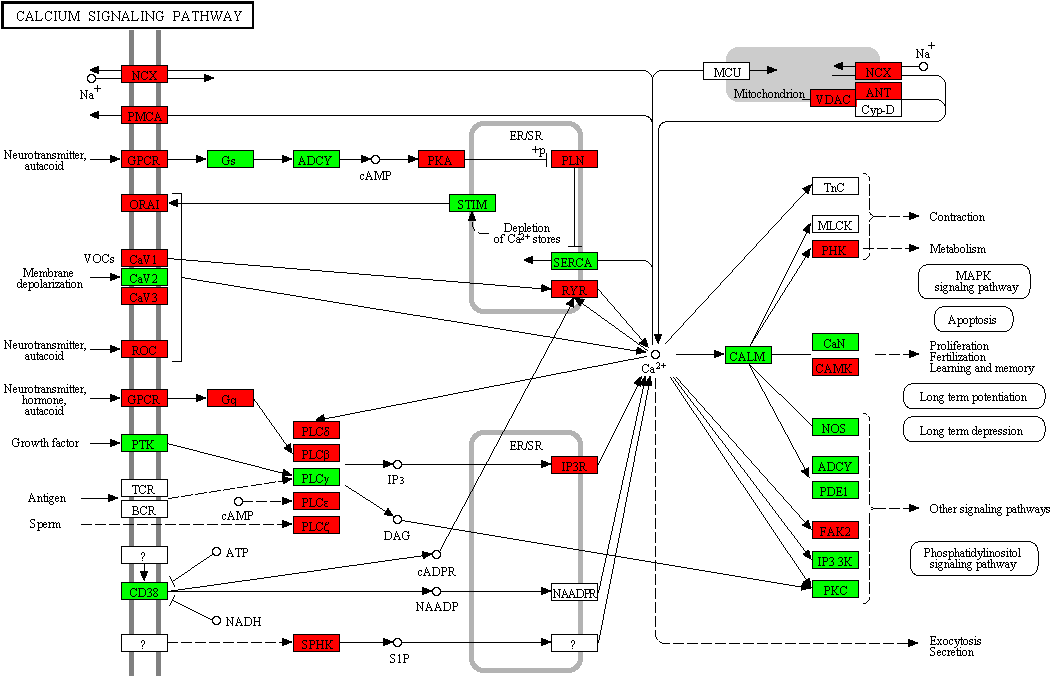


**Figure S3**


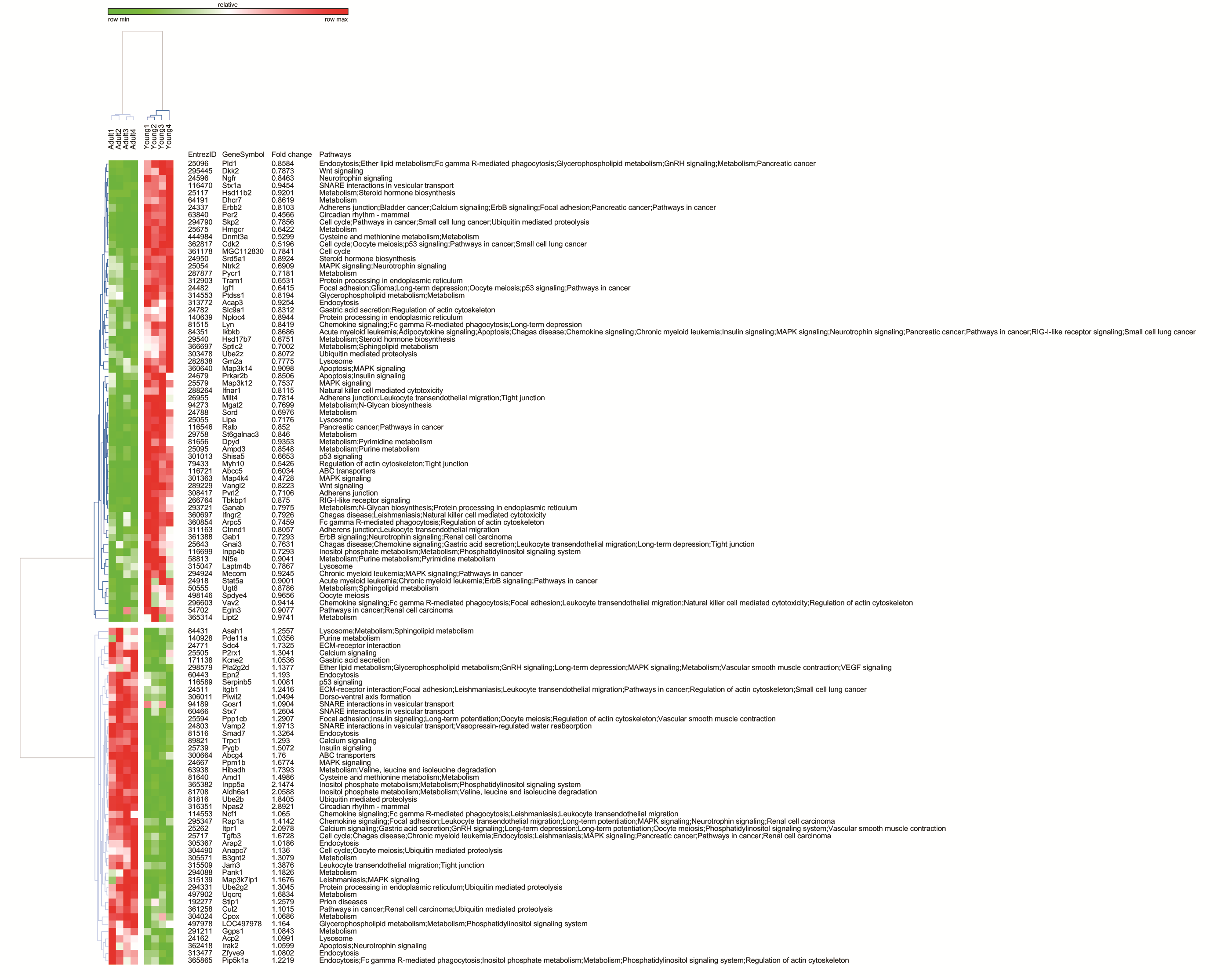


## Table S6

## *Common results between m- and mi-RNA microarrays data analysis*

**Figure 1** depicts the commonly identified candidates between significantly up-regulated genes (mRNA microarrays) and down-regulated miRNAs (**Figure 1a**), and significantly down-regulated genes (mRNA microarrays) versus up-regulated miRNAs (**Figure 1b**). These commonly identified candidates (**Figure 1**) are the representative members of oxidative phosphorylation, VLI degradation, TCA cycle, cardiac muscle contraction, cell cycle, focal adhesion, ECM-receptor interactions, DNA replication and steroid biosynthesis. The overlapping significantly enriched pathways between m- and mi-RNA microarrays are described in **Table 1**. **Table 2** represents the overlapping miRNAs found highly significantly enriched for their binding sites during bioinformatics analysis of m- and mi-RNA microarray data.

**Significantly up-regulated genes**

**p-regulated genes**

**target genes on down-regulated miRNAs**

**down-regulated miRNAs**

**Significantly down-regulated genes**

**down-regulated genes**

**Target genes on up-regulated miRNAs**

**up-regulated miRNAs**

**up-regulated miRNAs**


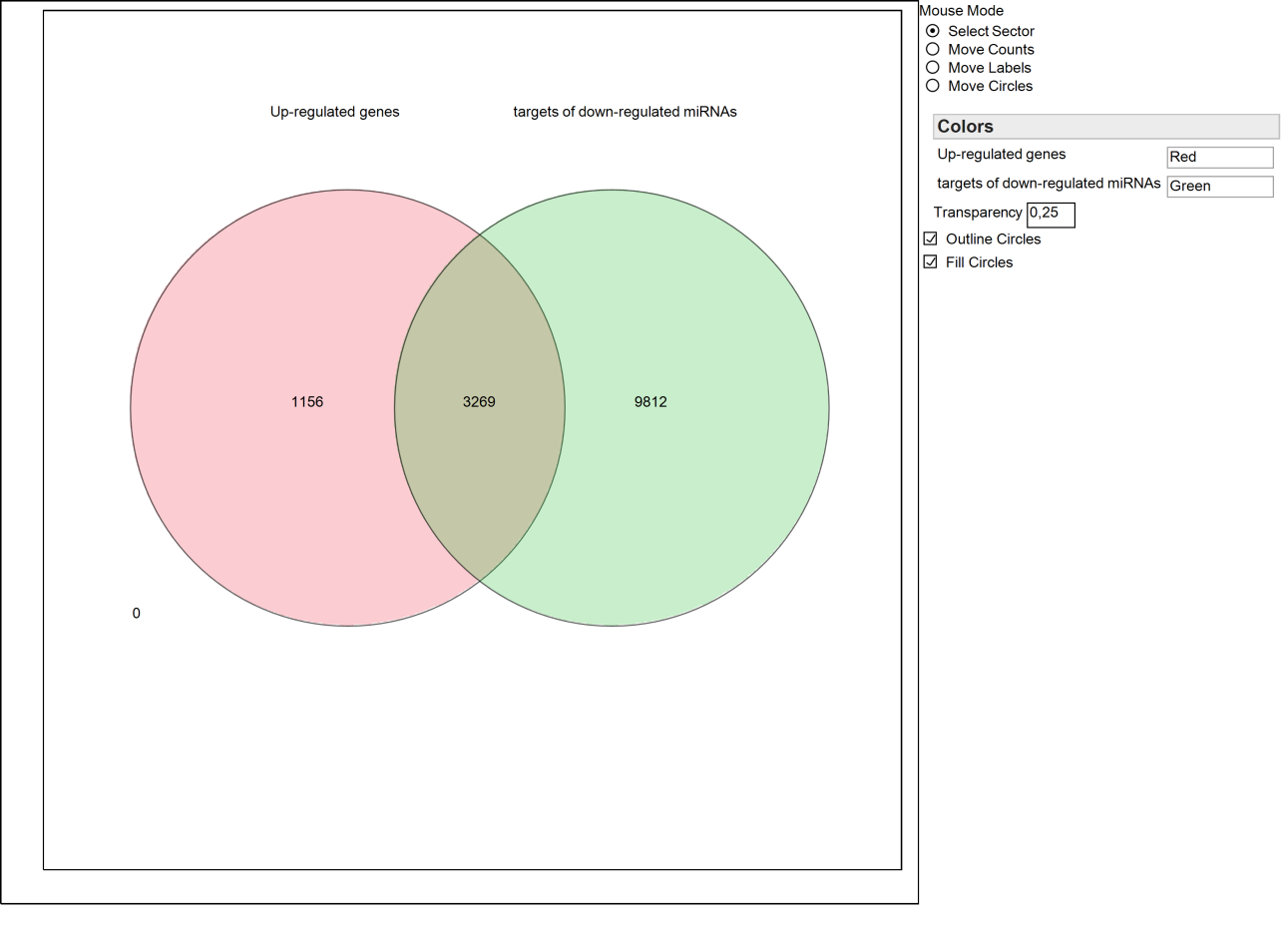

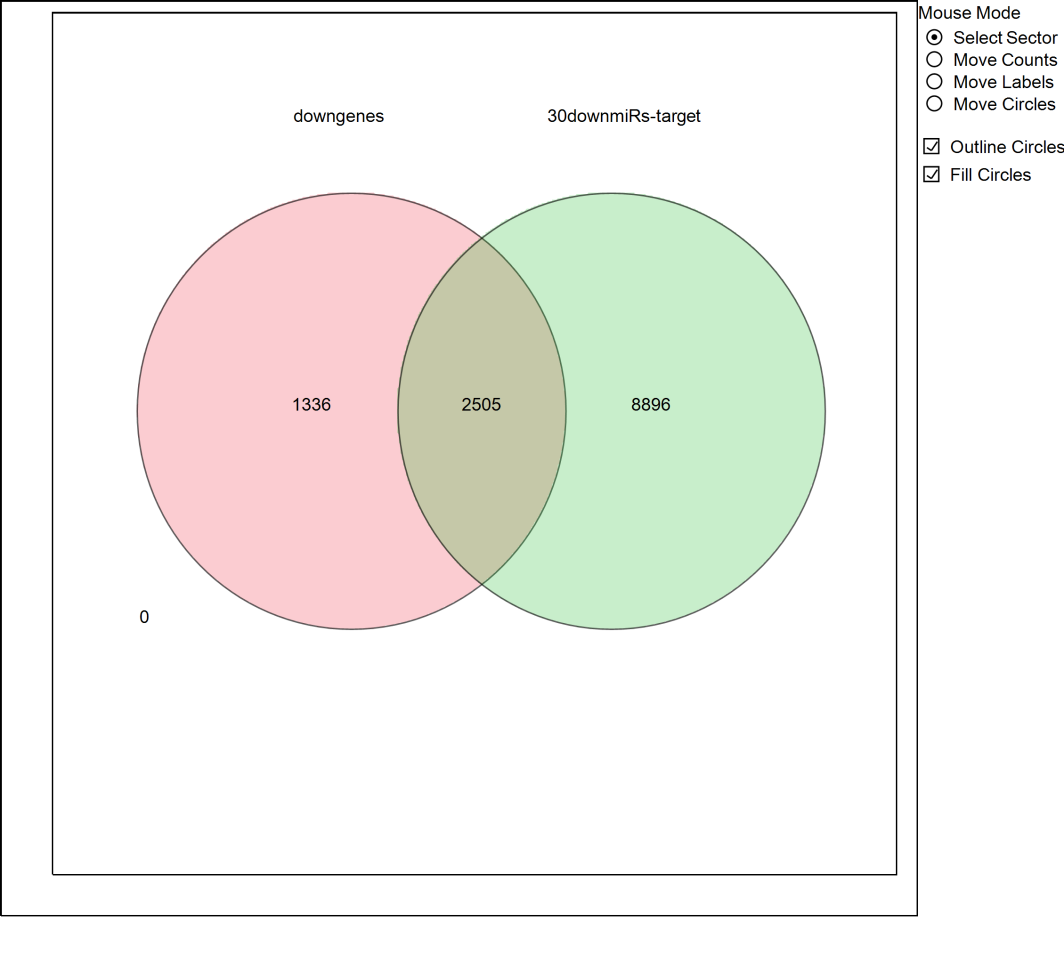


**(a)**

**(b)**

Figure 1. Common genes between m- and mi-RNAs microarrays data analysis. (a) Common candidates between significantly up-regulated genes and target-genes obtained on significantly down-regulated miRNAs and (b) common candidates between significantly down-regulated genes and target-genes predicted on significantly up-regulated miRNAs.

Disease6

Disease4

Disease1

Disease5

Disease3

Disease2

Control1

Control2

Control3

Control4

Disease6

**Table 1. Overlapping pathways (between m- and mi-RNA data) resulting from deregulated genes and putative target genes of dysregulated miRNAs.**

| *Overlapping pathways based on up-regulated genes and down-regulated miRNAs* |
| --- |
| Citrate cycle (TCA cycle), pentose phosphate pathway, valine, leucine and isoleucine degradation (VLI), pyruvate metabolism, propanoate metabolism, metabolic pathways, MAPK signaling, phosphatidylinositol signaling system, peroxisome, insulin signaling, leishmaniasis, chagas disease, hypertrophic cardiomyopathy (HCM) and dilated cardiomyopathy |
| ***Overlapping pathways based on down-regulated genes and up-regulated miRNAs*** |
| Steroid biosynthesis, N-Glycan biosynthesis, cell cycle, oocyte meiosis, protein processing in endoplasmic reticulum, notch signaling, axon guidance, focal adhesion, ECM-receptor interaction, adherens junction, progesterone-mediated oocyte maturation, bacterial invasion of epithelial cells and pathways in cancer |

Table 2. Common miRNAs found significantly enriched on m- and mi-RNA microarray data analysis.

| *Common miRNAs found enriched among up-regulated genes and miRNAs* |
| --- |
| rno-miR-193, -194, -1949, -195, -497, -331 and -374 |
| ***Common miRNAs found enriched among down-regulated genes and miRNAs*** |
| rno-miR-1224, -127, -130b, -134, -17-1-3p, -17-5p, -188, -18a, -19b, -20a, -20b-5p, -214,  -296, -298, -299, -300-3p, -322, -329, -341, -342-3p, -342-5p, -351, -369-5p, -370, -379,  -382, -409-5p, -411, -412, -431, -433, -434, -455*, -485, -487b, -494, -503, -539, -540, -665,  -667, -668, -672, -673, -678, -770 and -92a |
